# Membrane-cytoplasm translocation of annexin A4 is involved in the metastasis of colorectal carcinoma

**DOI:** 10.18632/aging.202793

**Published:** 2021-03-24

**Authors:** Ya Peng, Zhaoyu Zhang, Ailing Zhang, Changhong Liu, Yingnan Sun, Zixuan Peng, Yang Liu

**Affiliations:** 1 Hunan Provincial People's Hospital and the Affiliated Hunan Normal University, Changsha 410081, Hunan, China; 2 The Key Laboratory of Carcinogenesis of the Chinese Ministry of Health, The Key Laboratory of Carcinogenesis and Cancer Invasion of the Chinese Ministry of Education, Cancer Research Institute, Central South University, Changsha 410008, Hunan, China; 3 Hunan Provincial Tumor Hospital and the Affiliated Tumor Hospital of Xiangya Medical School, Central South University, Changsha 410013, Hunan, China

**Keywords:** annexin A4, calcium/calmodulin-dependent protein kinase II gamma, carbonic anhydrase, SUMO, membrane-cytoplasm translocation

## Abstract

Annexin A4 (ANXA4) is a Ca^2+^- and phospholipid-binding protein that belongs to the annexin family, which is involved in the development of multiple tumour types via NF-κB signalling. In this study, we verified the high expression and membrane-cytoplasm translocation of ANXA4 in colorectal carcinoma (CRC). Calcium/calmodulin-dependent protein kinase II gamma (CAMK2γ) was found to be important for high ANXA4 expression in CRC, whereas carbonic anhydrase (CA1) promoted ANXA4 aggregation in the cell membrane. An increased Ca^2+^ concentration attenuated the small ubiquitin-like modifier (SUMO) modification of cytoplasmic ANXA4 and ANXA4 stabilization, and relatively high expression of ANXA4 promoted CRC tumorigenesis and epithelial-mesenchymal transition (EMT).

## INTRODUCTION

Colorectal cancer (CRC) is the second most common cancer in women and the third most common cancer in men, causing more than 900,000 deaths worldwide annually [1]. In China, colon cancer represents the third most common cause of death (7.9%) among the deaths related to malignant neoplasms [2]. Despite the availability of comprehensive diagnostic and treatment methods, the average 5-year survival rate of colon cancer patients does not exceed 60%, and metastasis and recrudescence are still the main causes of death in CRC patients [1].

Annexin A4 (ANXA4) is a member of the annexin family and a Ca^2+^- and phospholipid-binding protein [3]. The ANXA4 gene is located on human chromosome 2q13 and encodes a 36-kDa protein that aggregates on the cell membrane and contains four annexin repeats with one Ca^2+^-binding site and five α-helices in each repeat, which can bind phospholipids in a Ca^2+^-dependent manner [3]. Studies have indicated that human ANXA4 is predominantly expressed in the secretory epithelia in the lungs, stomach, intestine, and kidneys [4]. ANXA4 accelerates membrane repair [5, 6], upregulates exocytosis [7–9], decreases mobility [10–12] and is involved in skin wound haemostasis [13], human heart failure [14] and tumours [15–21].

It is conceivable that ANXA4 is highly expressed in multiple tumour types and is an indicator for tumour development, invasion, chemo- resistance, and poor outcomes in cancer patients. ANXA4 biology may be a potential target for therapeutic intervention [3, 15–21], such as the Annexin A4-NF-κB feedback circuit that activates malignant cell behaviour and tumour growth in gallbladder cancer [18] and Annexin A4 fucosylation that enhances the interaction of ANXA4 with NF-kB p50 and promotes tumour progression in ovarian clear cell carcinoma [17]; toosendanin mediates cisplatin sensitization through targeting Annexin A4/ATP7A in non-small cell lung cancer cells [21].

Calcium/calmodulin-depende nt protein kinase II (CaMKII, also known as CAMK2γ) is a multifunctional serine/threonine protein kinase that transmits calcium signals in various cellular processes [22]. CAMK2γ is important in both haematopoietic malignancies and solid tumours [23, 24].

SUMOylation is a posttranslational modification that leads to diverse biological consequences. The addition of small ubiquitin-like modifier (SUMO) affects target proteins by altering their subcellular localization, enzymatic activity and/or protein-protein/protein-DNA interactions [25]. Three SUMO proteins are found in mammalian cells, with SUMO-2 and SUMO-3 sharing 97% sequence identity with one another but only approximately 50% identity with SUMO-1 [26]. Annexin A1 is regulated by domain cross-talk through posttranslational phosphorylation and SUMOylation [27].

Carbonic anhydrases (CAs) are a family of metalloenzymes that are found in almost every type of tissue. Carbonic anhydrases have 14 different isoforms, and the distribution patterns of the isoenzymes differ [28]. Silencing carbonic anhydrase I enhances the malignant potential of exosomes secreted by prostate tumour cells [29].

In this study, we verified the high expression and membrane-cytoplasm translocation of ANXA4 in colorectal carcinoma. Next, we further showed that CAMK2ɤ was indispensable for high ANXA4 expression in colorectal carcinoma and that the location of ANXA4 location in the cell membrane was Ca^2+^ dependent; CA1 could also promote ANXA4 aggregation in the cell membrane. Moreover, we also found that increasing the Ca^2+^ level decreased SUMOylation of cytoplasmic ANXA4 and that ANXA4 promoted CRC tumorigenesis and metastasis.

## RESULTS

### Cell membrane-cytoplasm translocation of ANXA4 in CRC

**Figure 1:**
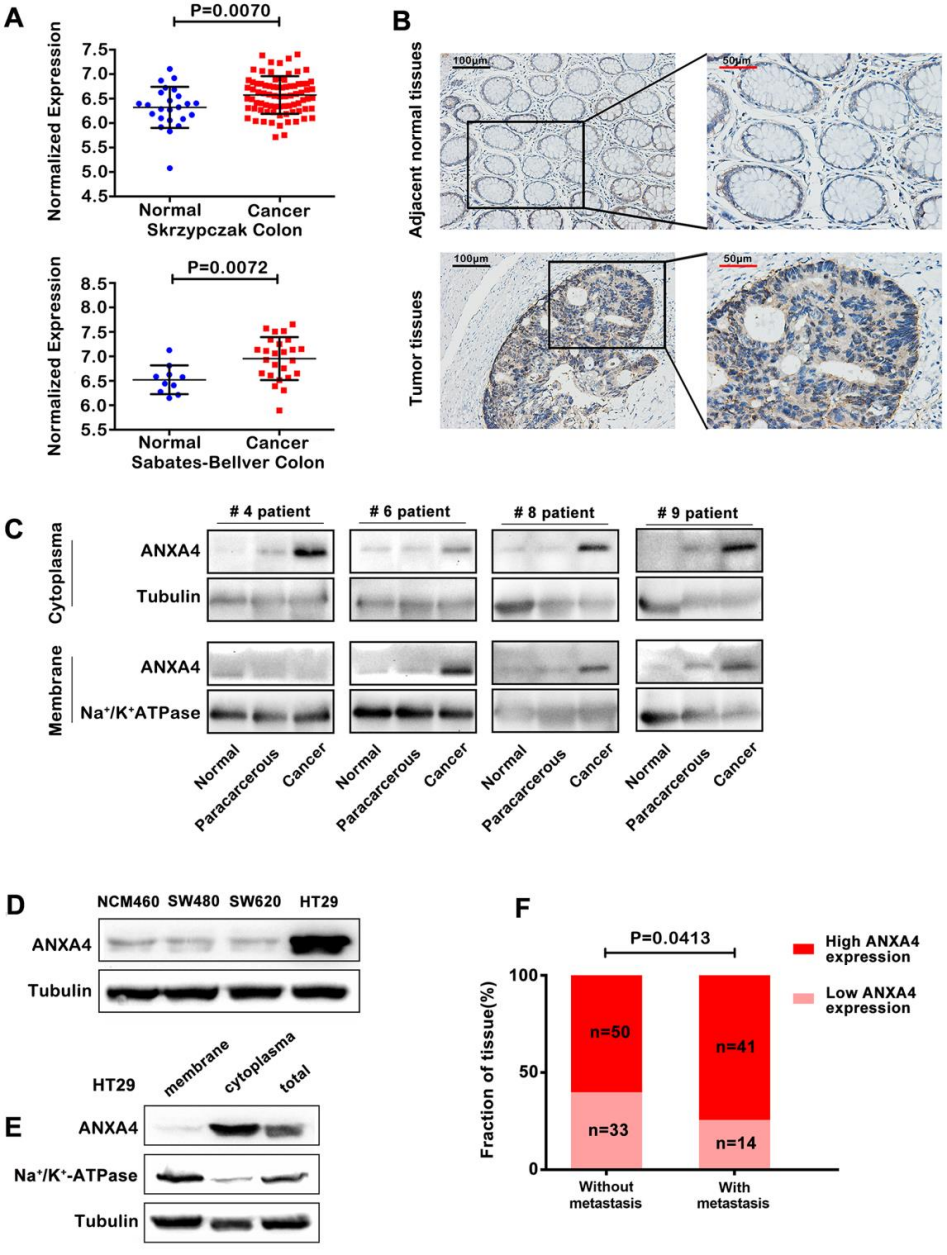
**Membrane-cytoplasm translocation of ANXA4 in CRCA.** (**A**) ANXA4 mRNA expression levels were significantly higher in colorectal cancer tissue samples than in normal tissue samples, as indicated by analysis of the Oncomine colorectal cancer dataset, Skrzypczak Colorectal dataset and Sabates-Bellver Colon dataset. (**B**) Representative images of the protein expression of ANXA4 in CRC and tumour-adjacent tissue samples (n=138) generated by immunohistochemistry. (**C**) Representative blots of the membrane and cytoplasmic protein expression of ANXA4 in CRC and tumour-adjacent tissue samples (n=19) generated by western blotting. Tubulin was used as the loading control for total protein; Na+/K+-ATPase was used as the loading control for cell membrane protein. (**D**) The expression of ANXA4 in normal colon cells (NCM460) and colorectal cancer cells (SW480, SW620, and HT-29 cells) determined by western blotting. Tubulin was used as the loading control for total protein. (**E**) The membrane and cytoplasmic protein expression levels of ANXA4 in HT-29 cells determined by western blotting. Na/K-ATPase was used as the loading control for cell membrane protein; Tubulin was used as the loading control for total protein. (**F**) Correlation analysis between high ANXA4 expression and metastasis in CRC patients (n=138) by chi-square test analysis (p<0.05).

Our previous studies have revealed that ANXA4 is highly expressed in CRC tissues and is a candidate biomarker in serum extracellular vesicles for detecting early-stage CRC [16]. Using Gene Expression Omnibus datasets (GEO) [GSE20916 (Poland), Skrzypczak Colorectal; GSE 8671 (Switzerland), and Sabates-Bellver Colon], we found that ANXA4 expression was significantly higher in colorectal cancer tissue than in the normal colonic mucosa (Figure 1A). As shown in Figure 1B, ANXA4 expression was strongly positive in CRC, and ANXA4 was located in both the membrane and cytoplasm in CRC tissues compared with the corresponding normal tissues, which were moderately stained and exhibited ANXA4 localized mainly in the plasma membrane (n=138). Furthermore, CRC patient tumour tissue samples, tumour-adjacent tissue samples and normal colon tissue samples (n=20) were used to examine the protein level and subcellular distribution of ANXA4 by western blotting of membrane-cytoplasm fractionated lysates. ANXA4 expression was higher in the CRC tissue samples than in the tumour-adjacent tissue samples and was detected in both the plasma membrane and cytosolic fractions in the CRC tissue samples, whereas ANXA4 expression was minimal in the normal tissue samples (Figure 1C). Comparison of endogenous ANXA4 levels between normal epithelial cells (NCM460) derived from the human colonic mucosa and three CRC cell lines (SW480, SW620, and HT-29) revealed that ANXA4 expression was significantly higher in HT-29 cells, which are derived from a highly metastatic CRC cell line, than in SW480 and SW620 cells [9] (Figure 1D) and that ANXA4 was mainly distributed in the cytoplasm of HT-29 cells (Figure 1E). Chi-square test analysis showed a positive correlation between high ANXA4 expression and lymph node metastasis in CRC patients (n=138) (Figure 1F).

### CAMK signalling affects the expression and location of ANXA4 in CRC

**Figure 2:**
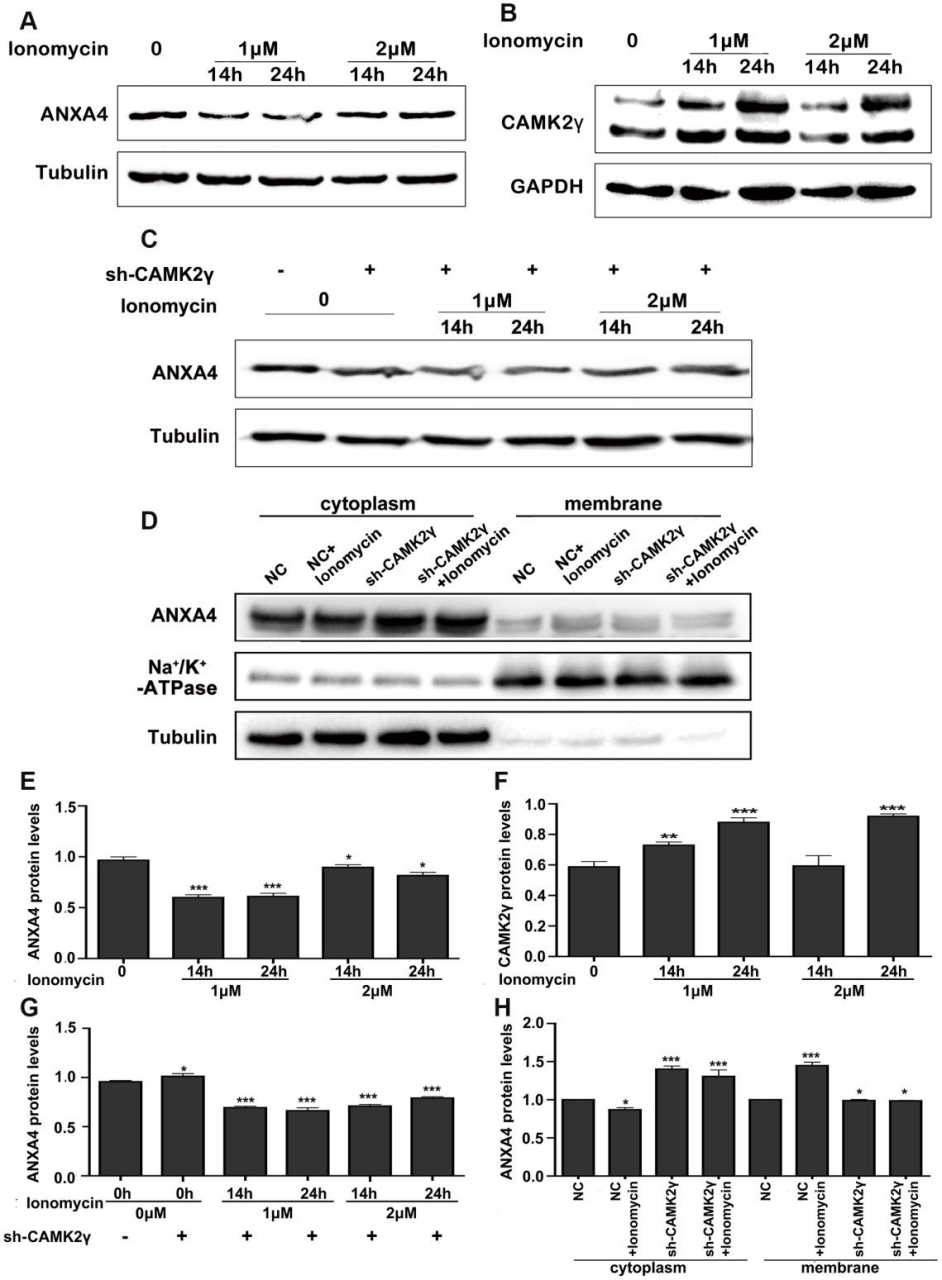
**Calcium regulated the expression and location of ANXA4.** (**A**) Time-course expression analysis of ANXA4 after the addition of the Ca2+ agonist ionomycin (1 μM or 2 μM) at 14 h and 24 h. Tubulin was used as the loading control for total protein. *p < 0.05; **p < 0.01; **p < 0.001. (**B**) Time-course expression analysis of CAMK2γ after the addition of the Ca^2+^ agonist ionomycin (1 μM or 2 μM) at 14 h and 24 h. Tubulin was used as the loading control for total protein. *p < 0.05; **p < 0.01; **p < 0.001. (**C**) Time-course expression analysis of ANXA4 in HT-29 cells transfected with CAMK2γ after the addition of the Ca^2+^ agonist ionomycin (1 μM or 2 μM) at 14 h and 24 h. Tubulin was used as the loading control for total protein. *p < 0.05; **p < 0.01; ***p < 0.001. (**D**) Membrane and cytoplasmic levels of ANXA4 in HT-29 cells transfected with CAMK2γ after the addition of the Ca^2+^ agonist ionomycin (1 μM) at 14 h. Na/K-ATPase was used as the loading control for cell membrane protein; Tubulin was used as the loading control for total protein. *p < 0.05; **p < 0.01; ***p < 0.001. (**E**–**H**) Relative quantitative of comparison of WB analysis mentioned in (**A**–**D**). *p < 0.05; **p < 0.01; ***p < 0.001.

ANXA4 contains conserved ANX repeat domains and binds phospholipids in a Ca2^+^-dependent manner [3]. High intracellular Ca2^+^ levels promote interactions between ANXA4 and the NF-κB p50 subunit, thus inhibiting the transcriptional activity of NF-κB [30]. We next wondered whether the calcium/CAMK signalling pathway has an effect on the regulation of ANXA4 in HT-29 cells. Western blotting was performed to detect ANXA4 expression after increasing the intracellular Ca2^+^ level by exogenously adding different concentrations of ionomycin (0 μM, 1 μM and 2 μM) for different times (2 h, 6 h, 14 h and 24 h) (Figure 2A, 2E). We found that 1~2 μM ionomycin decreased the expression of ANXA4 after treatment for 14 h (p<0.001) and 24 h (p<0.05).

Subsequently, we tested whether Ca^2+^ affects ANXA4 expression via CAMK. CaMKII is encoded by four different genes, α, β, γ and δ, and CAMK2γ is the major isoform in CRC cells [15]. We therefore evaluated whether Ca2+ decreases ANXA4 expression via CAMK2γ. Our results indicated that an increase in the intracellular Ca2^+^ level promoted CAMK2γ expression in HT-29 cells. As shown in Figure 2B, 2F, CAMK2γ expression increased nearly 2-fold after 1~2 μM ionomycin treatment for 24 h. We also transfected an shCAMK2γ plasmid into HT-29 cells and then exogenously added ionomycin (0 μM, 1 μM, or 2 μM) and found that interfering with CAMK2γ reversed the negative regulatory effect of increased Ca2^+^ levels induced by ionomycin on ANXA4 in HT-29 cells (Figure 2C, 2G). Results acquired after membrane-cytoplasm fractionation also clearly showed the translocation of ANXA4 into the membrane fraction after ionomycin treatment (Figure 2D, 2H). The above data suggest that CAMK2γ is important for high ANXA4 expression in HT-29 cells and that the localization of ANXA4 in the cell membrane is Ca2^+^ dependent.

### CA1 promoted ANXA4 aggregation in the cell membrane

**Figure 3:**
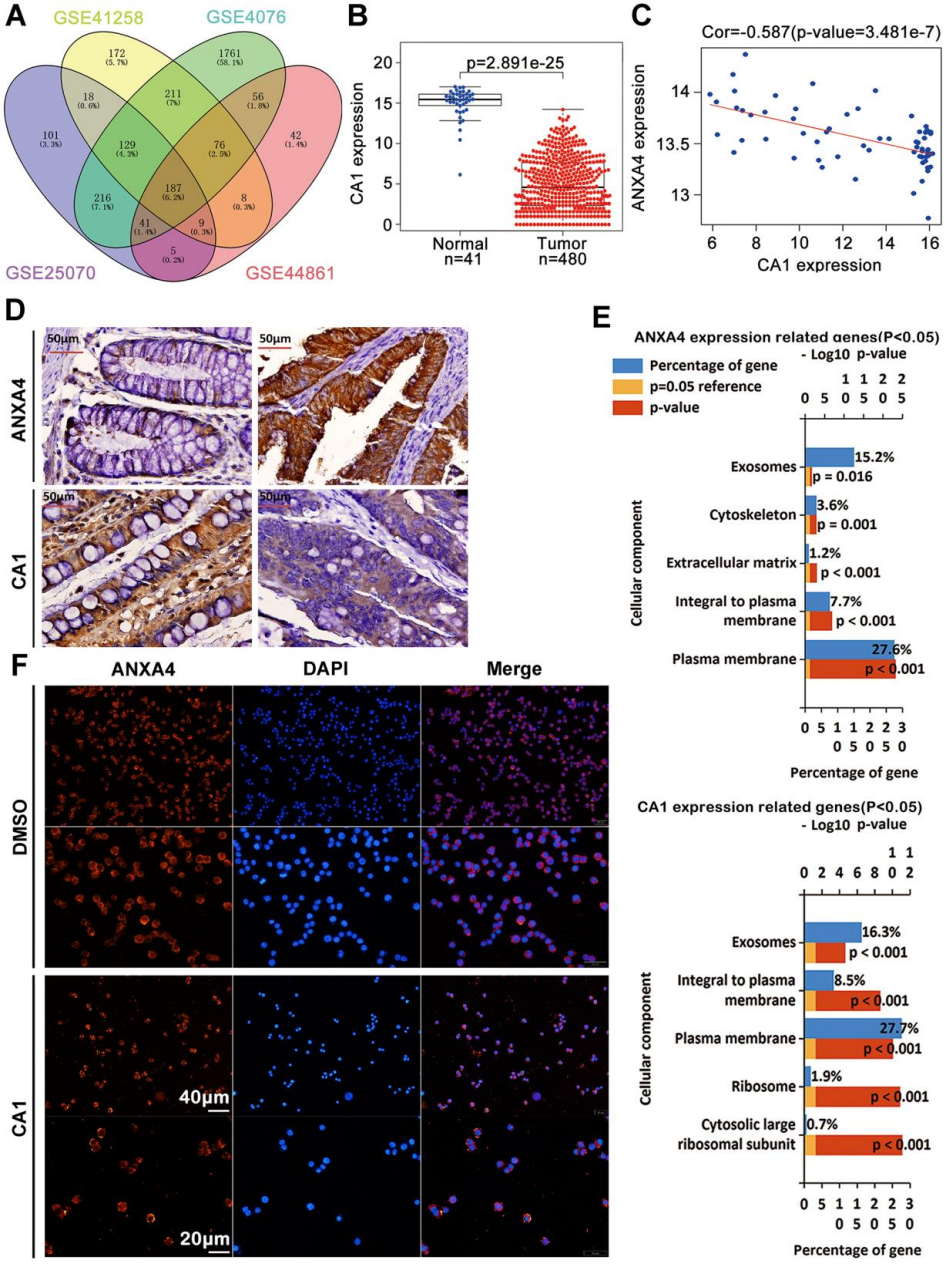
**CA1 promoted ANXA4 aggregation in the cell membrane.** (**A**) 187 commonly changed genes in CRC samples from the GSE 25070 (USA), GSE 41258 (Israel), GSE 44076 (Spain) and GSE 44861 (USA) datasets. (**B**) The CA1 mRNA expression level was significantly lower in colorectal cancer tissues than in normal tissues in the TCGA database. (**C**) Negative correlation analysis between ANXA4 and CA1 in the GSE 20916 and GSE 8671 datasets. (**D**) Decreased expression of CA1 and increased expression of ANXA4 in Chinese CRC samples, as determined by IHC (n=103). (**E**) Common cellular component enrichment results for CA1 and ANXA4 determined by GO analysis. (**F**) CA1 promoted ANXA4 aggregation in the cell membrane, as demonstrated by immunofluorescence imaging.

Our previous studies have revealed that CA1 is expressed at low levels and ANXA1 is highly expressed in clinical TNM stages I-IV by MALDI-TOF/TOF MS and immunohistochemical analyses of 103 CRC samples from Chinese patients [16]. In this study, we identified 187 commonly changed genes in CRC samples from the GSE 25070 (USA), GSE 41258 (Israel), GSE 44076 (Spain) and GSE 44861 (USA) datasets (Figure 3A), including CA1, which was also found to have low expression in a TCGA CRC dataset (Figure 3B). There was a negative correlation between CA1 and ANXA4 expression in GSE 20916 and GSE 8671 (Figure 3C), and this negative correlation between CA1 (low) and ANXA4 (high) expression was further verified in 103 Chinese CRC samples by IHC (Figure 3D). By mining TCGA data, we selected 50 upregulated and 50 downregulated genes correlated with ANXA4 or CA1 expression (p<0.05) to perform GO analysis (Figure 3E) and found that these genes are involved in the plasma membrane or integrated in the plasma membrane. This suggested that CA1 might be associated with the membrane-cytoplasm translocation of ANXA4. We used recombinant human CA1 to treat HT-29 cells and found that 1 μl CA1 promoted ANXA4 aggregation in the cell membrane (Figure 3F). These data suggest that the relatively low CA1 expression may be one of the reasons why ANXA4 is highly expressed and located in the cytoplasm in CRC tissues, but the mechanism is not particularly clear and needs further exploration.

### SUMOylation stabilized ANXA4 expression in the cytoplasm of CRC cells

Some studies have confirmed that the molecular expression and subcellular distribution of the Annexin A family can be regulated by SUMOylation [31]. However, the SUMOylation of ANXA4 and its effects on deallocation from the cell membrane to the plasma are still unknown, so we tested whether ANXA4 is a posttranslational modification target of SUMO.

**Figure 4:**
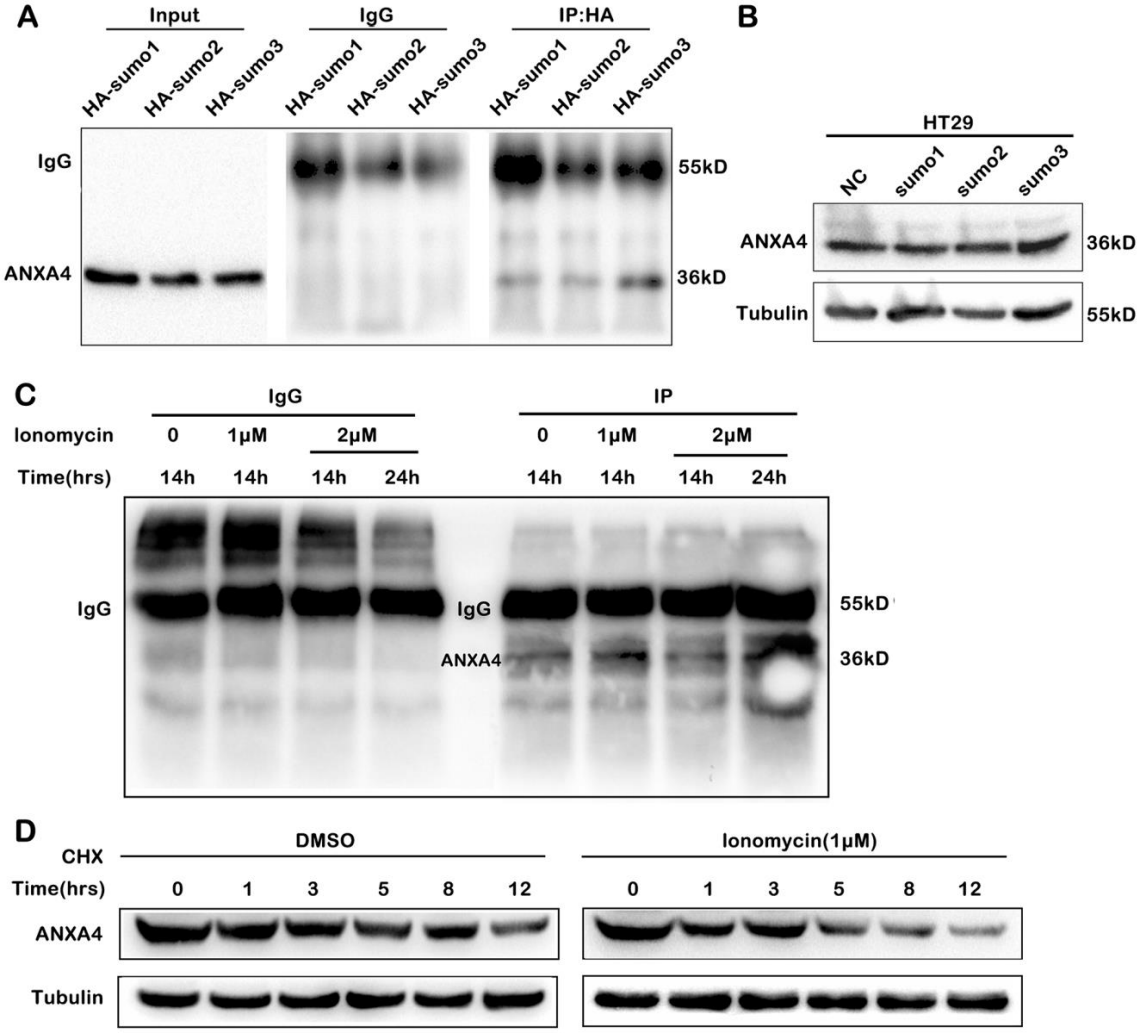
**SUMOylation of ANXA4 stabilized ANXA4 expression.** (**A**) Immunoprecipitation analysis of ANXA4 SUMOylation after cells were cotransfected with flag-ANXA4 and HA-SUMO1, HA-SUMO2, or HA-SUMO3 and then subjected to western blotting with an anti-HA antibody. (**B**) Western blot analysis of ANXA4 proteins in HT-29 cells after transfection with HA-sumo1, HA-sumo2 or HA-sumo3. Tubulin was used as the loading control. (**C**) Immunoprecipitation analysis of ANXA4 SUMOylation after cells were cotransfected with flag-ANXA4 and HA-SUMO1, treated with the Ca^2+^ agonist ionomycin (1 μM or 2 μM) at 14 h and 24 h and then subjected to western blotting with an anti-HA antibody. (**D**) Western blot analysis of the expression of ANXA4 in HT-29 cells after treatment with cycloheximide with the addition of the Ca^2+^ agonist ionomycin (1 μM).

We predicted potential SUMOylation sites in ANXA4 with bioinformatic software (SUMO plot TM Analysis and SUMOsp 2.0), and the three predicted conserved binding sites are found (data not shown). Then, we constructed the plasmids HA-SUMO1, HA-SUMO2, HA-SUMO3 and Flag-ANXA4; cotransfected HA-SUMO1, HA-SUMO2 or HA-SUMO3 and Flag-ANXA4 into HEK293T cells; and performed immunoprecipitation with anti-HA gel (Figure 4A). The results indicated that exogenous ANXA4 was mainly modified by SUMO1, SUMO2 and SUMO3; however, the transfection of an HA-SUMO plasmid did not upregulate ANXA4 expression (Figure 4B) or influence the membrane-cytoplasm translocation of ANXA4 in CRC cells (data not shown). These data indicated that SUMOylation was not associated with the membrane-cytoplasm translocation of ANXA4. However, SUMOylation of ANXA4 promoted ANXA4 stability, although the Ca2^+^ concentration increased (Figure 4C), whereas Ca2^+^ decreased the stabilization of ANXA4 (Figure 4D). This result is consistent with Ca2^+^ downregulating ANXA4 expression.

### High ANXA4 expression contributes to CRC tumorigenesis and metastasis

**Figure 5:**
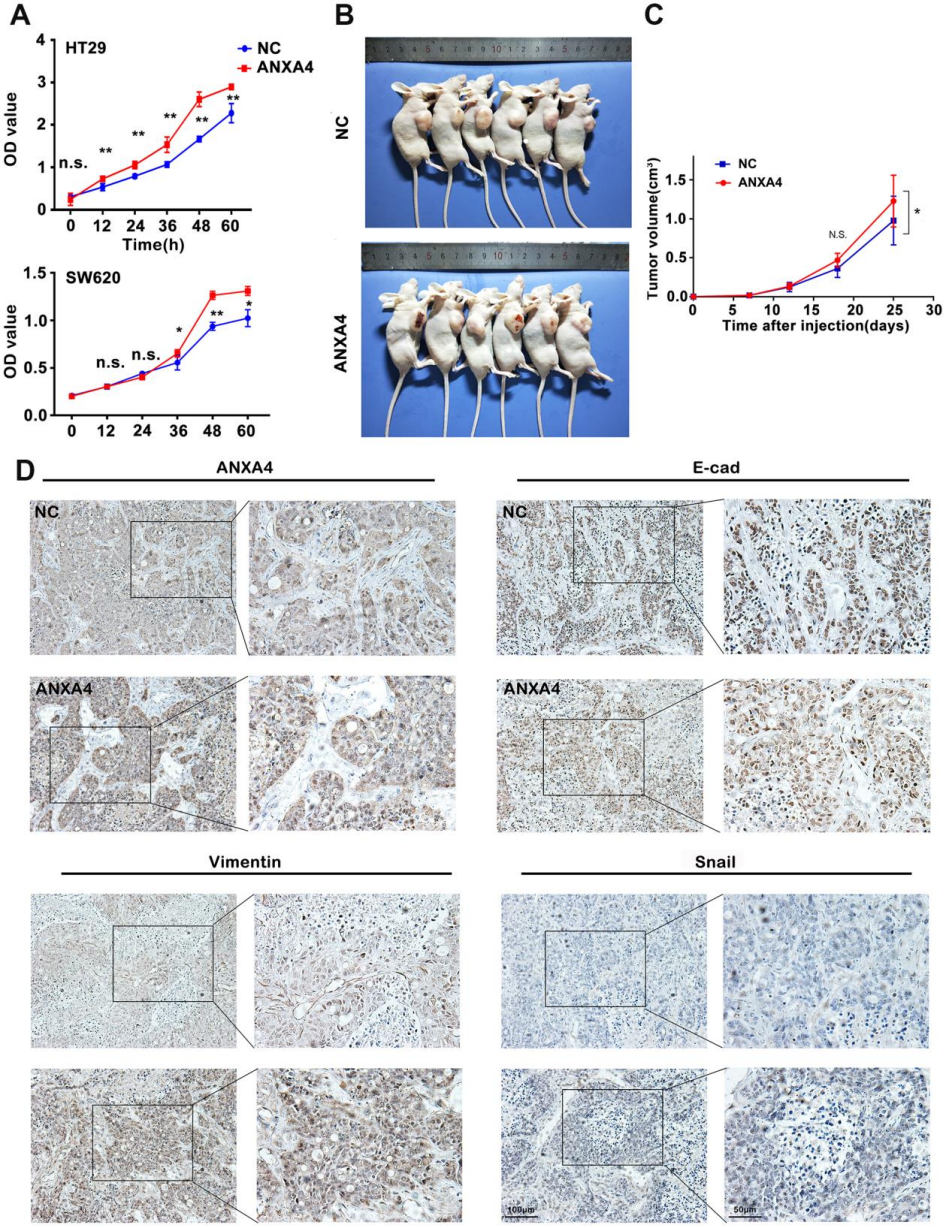
**ANXA4 promotes tumorigenesis and EMT in a xenograft model.** (**A**) CCK8 assay assessing the viability of HT-29 cells transfected with HA-ANXA4. The data are shown as the mean ± SEM of three independent experiments. *p < 0.05, **p<0.01. (**B**) HT-29 cells stably transfected with ANXA4 were subcutaneously injected into six nude mice (2×10^6^/120 μl per mouse). After 25 days, the mice were sacrificed, and the tumours were removed. (**C**) Tumour growth curves of the xenograft tumours (n=6). Tumour volume was calculated at different time points (7, 12, 18, and 25 days) using the formula V=(a×b2)/2. *p < 0.05. (**D**) Protein expression levels of ANXA4 and the EMT-related molecules E-cadherin, Vimentin, and Snail in tumours determined by IHC.

We further explored the impacts of ANXA4 on the proliferation and metastatic potential of HT-29 cells. Overexpression of ANXA4 resulted in an increase in viability, as measured by using a CCK8 assay (Figure 5A). A wound-healing assay and Matrigel invasion assay showed that ANXA4 promoted the cell migration and invasive potential of HT-29 cells (data not shown). In addition, HT-29 cells transfected with ANXA4-overexpression or empty vectors were injected subcutaneously into nude mice, and tumour volume was evaluated at 7, 12, 18, and 25 days. The *in vivo* xenograft model indicated that the ANXA4 overexpression groups had significantly higher tumourigenicity with obvious haemorrhagic necrosis on the tumour surface than the control group (n=6, n1=3/6 VS n2=0/6) (Figure 5B, 5C). IHC identified the protein expression levels of ANXA4 and the EMT signalling pathway factors E-cadherin, Vimentin and Snail in tumours. Higher ANXA4 expression was accompanied by lower E-cadherin expression and higher Vimentin and Snail expression (Figure 5D).

Although we did not find tumour metastasis in our subcutaneous xenograft tumour model because of time limitations (25 days), we assessed the relationships between clinicopathological characteristics and ANXA4 expression levels by immunohistochemical staining of 138 CRC samples and corresponding tumour-adjacent tissue samples. Higher expression of ANXA4 was correlated with tumour differentiation (p<0.001) and metastasis (p<0.05) (Figure 1F), but it was not correlated with sex, age, tumour location or tumour size.

### ANXA4 caused the downregulation of the stromal response to promote EMT in CRC

**Figure 6:**
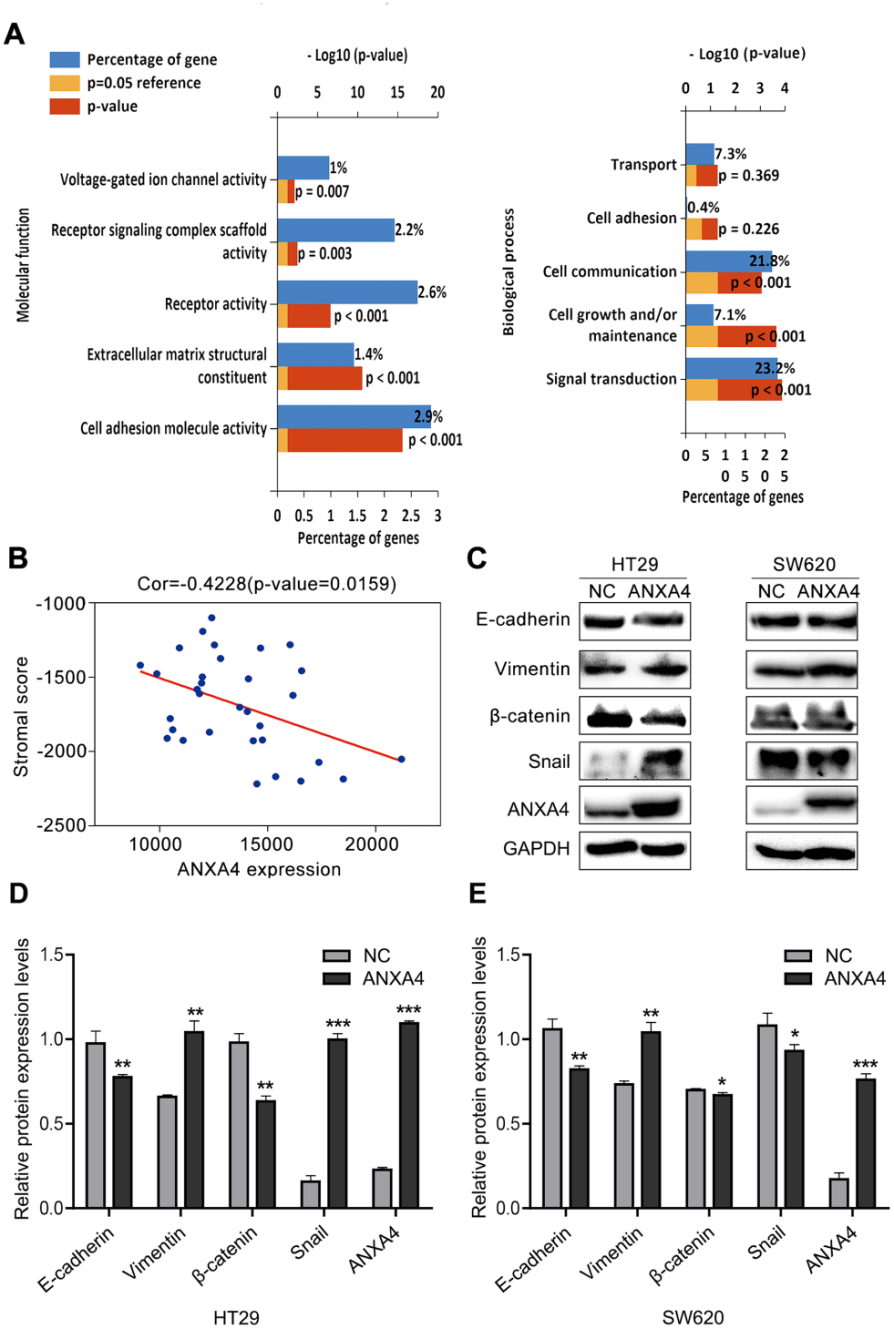
**ANXA4 downregulated the stromal response in CRC.** (**A**) Common molecular function and biological process enrichment terms of ANXA4 determined by GO analysis. (**B**) Negative correlation analysis between ANXA4 and the stromal score. (**C**) Effects of ANXA4 overexpression on the EMT-related molecules E-cadherin, Vimentin, β-catenin and Snail. GAPDH was used as the loading control for total protein. (**D**, **E**) Relative quantitative analysis of E-cadherin, Vimentin, β-catenin and Snail expression in HT-29 cells (**D**) and SW620 cells (**E**). *p < 0.05; **p < 0.01; ***p < 0.001.

As shown in Figure 3E, GO analysis was focused on the molecular function and biological pathway categories for ANXA4 expression-related genes, and we found that ANXA4 was mainly involved in receptor signalling complex scaffold activity, extracellular matrix structural constituent, cell adherin molecule activity, integrin family cell surface interaction and epithelial-mesenchymal transition (Figure 6A). Based on the ESTIMATE algorithm, stromal scores ranged from -1307.73 to 1112.11, and immune scores were distributed between -1097.42 and 1759.18. We found that ANXA4 did not change immune scores, but ANXA4 decreased stromal scores (Figure 6B), which was consistent with the GO analysis results. The above analysis implies that ANXA4 may be involved in CRC metastasis.

We further investigated the effects of ANXA4 on EMT-related genes (E-cadherin, Vimentin, β-catenin, and Snail) in CRC cells by transfecting ANXA4-carrying plasmids into SW620 or HT-29 cells and evaluating the cells by western blotting. We found that ANXA4 increased the expression levels of vimentin and Snail 1 but decreased the expression of E-cadherin and β-catenin (Figure 6C–6E). The same results were obtained in the context of ANXA4 overexpression in the subcutaneously transplanted tumour nude mouse model (Figure 5D).

## DISCUSSION

Annexin A4 (ANXA4) can reversibly bind to membrane phospholipids in a calcium-dependent manner and is involved in regulating tumour cell proliferation, apoptosis, adhesion, invasion, metastasis, and chemotherapy resistance, as well as other biological processes [3, 15–21]. In the current study, western blot and immunohistochemistry analyses of ANXA4 expression and localization indicated that ANXA4 was highly expressed and located in the cell membrane and cytoplasm in CRC tissue compared to normal colon tissue and that the quantity of ANXA4 in the cytoplasm was obviously greater than that in the nucleus.

We next explored the mechanism underlying the cell membrane to cytoplasm shift in ANXA4 localization in CRC. Gaudio E et al. [32] reported that the Fhit protein can interact with ANXA4 and that Fhit overexpression prevents ANXA4 translocation from the cytosol to the plasma membrane in A549 lung cancer cells treated with paclitaxel. Our results indicated that under stimulation with the Ca2^+^ activator ionomycin, increasing Ca2^+^ concentrations decreased the expression of ANXA4 in HT-29 cells but promoted ANXA4 assembly in the cell membrane. CAMK2γ in intestinal epithelial cells modulates colitis-associated colorectal carcinogenesis by enhancing STAT3 activation [23]. CAMK2γ antagonizes growth factor- or insulin-induced mTORC1 activation by inhibiting IRS1/AKT signalling [24]. Our results indicated that Ca2^+^ stimulation alone decreased the stabilization of ANXA4 (Figure 4D) to limit ANXA4 expression but CAMK2γ was indispensable for high ANXA4 expression in HT-29 cells and ANXA4 localization in the cell membrane in a Ca2^+^-dependent manner. Moreover, we found that relatively low CA1 expression was accompanied by relatively high ANXA4 expression and promoted ANXA4 localization in the cytoplasm in CRC.

Small ubiquitin-like modifier, including SUMO 1-4 in humans, is a reversible protein modifier. SUMO modification (SUMOylation) is a newly discovered post-translational regulatory mechanism that plays critical roles in a variety of cellular processes by regulating the stability or translocation of target proteins [31]. In particular, SUMOylation can impact the subcellular distributions of proteins; for example, SUMOylated KHSRP is more likely to translocate from the nucleus to the cytoplasm than is unmodified KHSRP in glioblastoma multiform cells [33]. In colorectal carcinoma, Ca2^+^ did not affect the interaction between SUMO1 and ANXA4, and SUMOylation did not affect the membrane-cytoplasm translocation of ANXA4. After ANXA4 was translocated into the cytoplasm, it was further modified by SUMOylation to make it more stable, supporting its involvement in CRC tumorigenesis and progression.

## MATERIALS AND METHODS

### Patients and tissue samples

Samples from one hundred forty-three patients diagnosed with CRC were obtained from the Hunan Provincial Tumor Hospital and the affiliated tumour hospital of Xiangya Medical School. For all cases, pathological and clinical follow-up data were collected after obtaining ethical approval from the China Ethical Review Committee. CRC was diagnosed based on clinical symptoms, proctoscopy and biopsy.

### Cell culture and transfection

SW480, SW620, HT-29 and HEK293 cells were obtained from the Cell Center of Peking Union Medical College (Beijing, China) and were maintained in RPMI-1640 medium (Gibco) containing 10% foetal bovine serum (Sigma) and antibiotics (100 μg/ml penicillin and streptomycin) in 5% CO2 in a 37° C incubator.

The SW620 cell line is derived from a lymph node metastasis of the primary tumour from which the SW480 cell line is derived. HT 29 cells are derived from another primary colorectal adenocarcinoma.

For calcium stimulation, ionomycin (MCE) was dissolved in DMSO, added to medium at a final concentration of 1 or 2 μM and used for stimulation for 8 or 16 h. Cycloheximide (CHX, MCE) was added to culture medium at 100 μM and applied for 1, 3, 5, 8 or 12 h.

HA-SUMO1, HA-SUMO2 and HA-SUMO3 were purchased from Addgene Company. pcDNA3.1-shRNA-CAMK2G, pcDNA3.1-Flag-ANXA4 and pcDNA3.1-empty control were purchased from Integrated Biotech Solutions Company. All the plasmids were confirmed by sequencing. Cell transfection was performed using Lipofectamine 3000 (Invitrogen) according to the manufacturer’s instructions. Recombinant human carbonic anhydrase-1 (CA1) was purchased from ProSpec Company (China, ENZ-462).

### Intracellular Ca^2+^ level increase

Ionomycin was added to HT-29 cells at different concentrations (0 μM, 1 μM and 2 μM) for different times (2 h, 6 h, 14 h and 24 h) to increase the intracellular Ca^2+^ level. HT-29 cell proteins were extracted for immunoblotting.

### Western blotting

Proteins were extracted from cell lines or tissue and lysed in GLB^+^ supplemented with a protease inhibitor cocktail (Bimake) for 20 min on ice, followed by centrifugation (12,000 rpm, 4° C, 15 min). Membrane and cytoplasmic proteins were prepared with the Mem-PER™ Plus Membrane Protein Extraction Kit (Thermo, 89842). Then, 25-60 μg of protein extracts were loaded onto 8-12% electrophoresis gels and transferred to membranes. The membranes were blocked with 5% non-fat milk and subsequently incubated with primary antibodies. The antibodies used were anti-ANXA4 (Proteintech), anti-CAMK2G (Proteintech), anti-HA (Proteintech), anti-FLAG (Sigma), anti-E-cad (Cell Signaling Technology), anti-N-cad (Cell Signaling Technology), anti-Vimentin (Cell Signaling Technology), anti-Snail 1 (Proteintech), anti-β-catenin (Proteintech), anti-Tubulin (Proteintech), anti-GAPDH (Proteintech) and anti-Na+-K+-ATPase (Santa Cruz).

### Analysis of SUMO-modified ANXA4

In total, 1 × 10^7^ HEK-293 cells and HT-29 cells were plated in 10-cm dishes and transfected with 10 μg of f1ag-ANXA4 and HA-SUMO1, HA-SUMO2, or HA-SUMO3. After 48 h of co-transfection, the cells were lysed in RIPA buffer. Next, we conducted IP experiments. The first antibody we used was anti-HA.

### Immunoprecipitation

HA-SUMO1, HA-SUMO2 or HA-SUMO3 was co-transfected with Flag-ANXA4 into HEK-293 cells and immunoprecipitated with anti-HA gel. ANXA4 immunoprecipitation from replicating chromatin under denaturing conditions was performed by resuspending the chromatin pellet in chromatin preparation buffer (CPB) (20 mM HEPES, pH 7.6; 100 mM KCl; 2% sucrose; and 5 mM MgCl2) supplemented with 1% SDS, followed by 10-fold dilution in CPB supplemented with 1% Triton X-100. The samples were then incubated with 500 ng of anti-ANXA4 antibodies with agitation at 4° C for 45 min, followed by the addition of protein A Dynabeads and incubation at 4° C for 1 h.

### Immunohistochemistry

CRC and normal colorectal tissue samples were sectioned into 7-mm-thick slides and incubated with appropriate antibodies at 4° C in a humidified container. A non-biotin horseradish peroxidase detection system and DAB substrate (Ultra Sensitive TM SP IHC Kit, Biotechnologies) were used for staining. A semi-quantitative scoring system was used to evaluate the ANXA4 protein levels, which considered both the intensity and extent. The ANXA4 protein levels were scored as follows: 0 (negative), 1 (weak), 2 (moderate), and 3 (strong). The scores were evaluated independently by two experienced pathologists blinded to the patients’ clinical characteristics and outcomes, and the average ANXA4 IHC score was recorded as low (score<1) or high (score>2).

### Wound healing and cell invasion assays

Sixteen hours after transfection of an ANXA4-carrying plasmid, cells reached 70% confluence in 6-well plates. The cell layers were scratched using a 10-μl tip to form wound gaps, washed with PBS twice and cultured in serum-free medium. The wound gaps were imaged at different time points and analysed by measuring the distance of migrating cells in five different areas for each wound.

Transwell inserts (8-μm pore, Corning) were precoated with a 20-μL mixture of Matrigel (BD Biosciences) and 1640 medium (Sangon Biotech) at a ratio of 1:1 for 1 hour at 37° C. HT-29 cells were transfected with ANXA4/empty control vector for 36 h, and then 200 μl of cell suspension (105 cells) was placed into the upper chambers with 2% FBS 1640 medium. After 28 h of incubation, the cells inside the upper chamber were removed with cotton swabs. The invaded cells on the lower membrane surface were fixed and then stained with 10% crystal violet. Four randomly selected fields in each well were counted.

### Xenograft tumour model

All animal experiments were approved by the Animal Care and Use Committee of Central South University. For subcutaneous tumour formation, cells (2×10^6^) in 100 μl of serum-free medium were injected subcutaneously into the left flank of nude mice. A total of 12 mice were used for the intracranial xenograft tumour model, which included the ANXA4 overexpression groups and control groups. Six mice in each group were sacrificed after 25 days for IHC. Tumour volumes were determined according to the following formula: A×B2/2, where A is the largest diameter and B is the diameter perpendicular to A.

### Statistical analysis

All experiments were analysed with GraphPad Prism 5 (La Jolla, CA, USA). Inter-group differences were tested using Student’s t-test or one-way ANOVA. Correlation analysis was performed with Spearman’s rank test and the chi-square test. Data are expressed as the mean ± SEM. of at least three independent experiments. A probability value (p) <0.05 was considered statistically significant.
